# Use of a Spinal Thermal Massage Device for Anti-oxidative Function and Pain Alleviation

**DOI:** 10.3389/fpubh.2020.00493

**Published:** 2020-09-16

**Authors:** Ka-Eun Kim, Jeong-Sook Park, Il-Young Cho, Yong-Soon Yoon, Soon-Kwon Park, Sang-Yun Nam

**Affiliations:** ^1^College of Medical Sciences, Jeonju University, Jeonju-si, South Korea; ^2^Department of Nursing, Nambu University, Gwangju, South Korea; ^3^College of Medical Sciences, Jeonju University, Jeonju-si, South Korea; ^4^Department of Rehabilitation Medicine, Presbyterian (Jesus) Medical Center, Jeonju-si, South Korea; ^5^College of Social Sciences, Jeonju University, Jeonju-si, South Korea; ^6^College of Medical Sciences, Jeonju University, Jeonju-si, South Korea

**Keywords:** spinal thermal massage device, pain, superoxide dismutase, glutathione peroxidase, catalase

## Abstract

**Background:** Elderly people are vulnerable to a variety of diseases, including chronic pain, which reduces their levels of physical fitness. Thermal massage has been shown to relieve pain and activate antioxidant enzymes. The objective of this study was to determine whether thermal massaging of the spinal column can reduce muscle pain and induce antioxidant function.

**Methods:** This study included participants aged ≥60 years with lower back pain. The participants were assigned to either an experimental group who received spinal column thermal massage and standard rehabilitative treatment or a control group who received standard rehabilitative treatment only. Data from a total of 116 participants (61 and 55 in the control and experimental groups, respectively) were used for analysis. Participants were assessed before treatment and at 4 (POST1) and 8 weeks (POST2) post-treatment, using a pain numeric rating scale (PNRS) and the Roland and Morris Disability Questionnaire (RMDQ), and by measuring the serum levels of superoxide dismutase (SOD), serum glutathione-peroxidase (GPx), and serum catalase (CAT).

**Results:** The extent of pain reduction, as measured by the PNRS, was greater in the experimental group. The RMDQ score in the control group decreased at POST1, but the decrease was not maintained at POST2, whereas the decrease in POST1 in the experimental group continued until POST2. SOD concentrations were significantly higher in the experimental group at POST1 and POST2, and GPx levels were significantly higher in the experimental group at POST2; however, there were no changes in CAT concentrations. Incidentally, there was a significant correlation between antioxidant activity and pain perception in the experimental group.

**Conclusions:** The study findings suggest that spinal column thermal massage reduces pain more effectively, improves self-reported levels of disability, and increases the antioxidant enzyme levels. Thermal massage may, therefore, be useful in the prevention and treatment of diseases associated with oxidation.

## Introduction

Elderly people are vulnerable to various diseases, including chronic pain, and often experience a sudden decline in fitness, limiting them to low-level physical activity (1). Among various theories regarding the causes of aging, the theory involving oxidative stress is a widely accepted one. With aging, the levels of antioxidant enzymes in the body decrease and the total blood antioxidant activity that can respond to oxidative stress decreases gradually (2, 3). This subsequently leads to an increase in the production of reactive oxygen species (ROS), which is a major cause of geriatric diseases and other diseases responsible for chronic pain (4, 5).

While various factors can cause pain, inflammation by ROS is of particular importance. Excessive amounts of ROS, which are produced naturally by metabolic processes, may cause cell damage by oxidation (6, 7). In such events, the cell membrane, DNA, and other cellular structures may become damaged. Consequently, the cells become dysfunctional or mutated depending on the extent of damage, and pain may occur due to the substances generated during this process (8).

Recently, studies have identified the intracellular mechanisms and physiological effects triggered by massage. Specifically, studies have reported that the levels of cytokines, including interleukin-6 (IL-6) and tumor necrosis factor-alpha (TNF-α), which increase following the inflammatory response and micro-rupture of tissues due to muscle injury or strenuous exercise, and nuclear factor-κB (NF-κB), a transcription factor that activates these cytokines, were reduced significantly by massage (9, 10). NF-κB is especially sensitive to oxidation and is known to activate inflammatory cytokines when it is stimulated by ROS (11). Taken together, the results from previous studies suggest that massage activates antioxidant functions and alleviates inflammation and pain through the reduction of NF-κB, IL-6, and TNF-α levels. When ROS activate the signaling systems, such as NF-κB or mitogen-activated protein kinase, or disturb their homeostasis, cells may proliferate abnormally or mutate (12). Cells possess various antioxidant enzymes and chemicals for eliminating ROS (13). The antioxidant enzymes that protect the body by removing free radicals generated by oxidative stress include superoxide dismutase (SOD), catalase (CAT), glutathione-peroxidase (GPx), and glutathione S-transferase (GST) (14, 15). Karabulut et al. reported that the level of antioxidant enzymes was increased after a massage intervention was applied to obese elderly women (16). Moreover, results from a randomized double-blind study revealed that the pain experienced by patients with chronic pancreatitis was reduced after they were treated with antioxidants (17).

When heat is applied to human tissues, the increase in tissue temperature is accompanied by physiological responses (18), including an increase in collagen fibril extensibility, blood flow, and cell permeability, and a reduction in muscle spasms and pain (19, 20).

Recently, home healthcare devices, such as spinal thermal massage (STM) devices, with massage and heating functions, have been developed and are commercially available. Among these devices, some products have already received food and drug administration (FDA) approval from various countries, to be used as medical devices for reducing muscle pain. However, few studies have evaluated the pain-alleviating effects of these medical devices. Accordingly, the present study aimed to clinically identify the pain-alleviating effects of a STM program, and to determine whether these programs induce changes in the antioxidant enzyme activity.

## Methods

### Participants

This study followed the principles and recommendations of the Helsinki Declaration, and the protocol was reviewed and approved by the Institutional Review Board (IRBN. 2017-06-022). A total of 140 adults (≥60 years of age) who had experienced lower back, shoulder, knee, hip, and/or neck pain for at least 3 months were recruited for this study, and we obtained the written informed consent from them. We evaluated the data obtained from their medical records and interviews prior to the study to determine if the candidates were eligible to participate in this study.

Candidates with any one of the following conditions were excluded from the study: diseases of the immune system, pacemaker or an electronic implanted device, malignant tumor, spinal infection, thrombosis, skin disease or skin hypersensitivity, spinal deformity or scoliosis with Cobb's angle ≥ 20°, osteoporosis or history of spinal fracture due to osteoporosis, high risk of spinal fracture, myopathy, or spinal instability (failed back syndrome) following spinal surgery. We also excluded anyone who was determined to be unfit for the study by the researcher. Ten candidates were eliminated during this screening phase.

**Figure 1:**
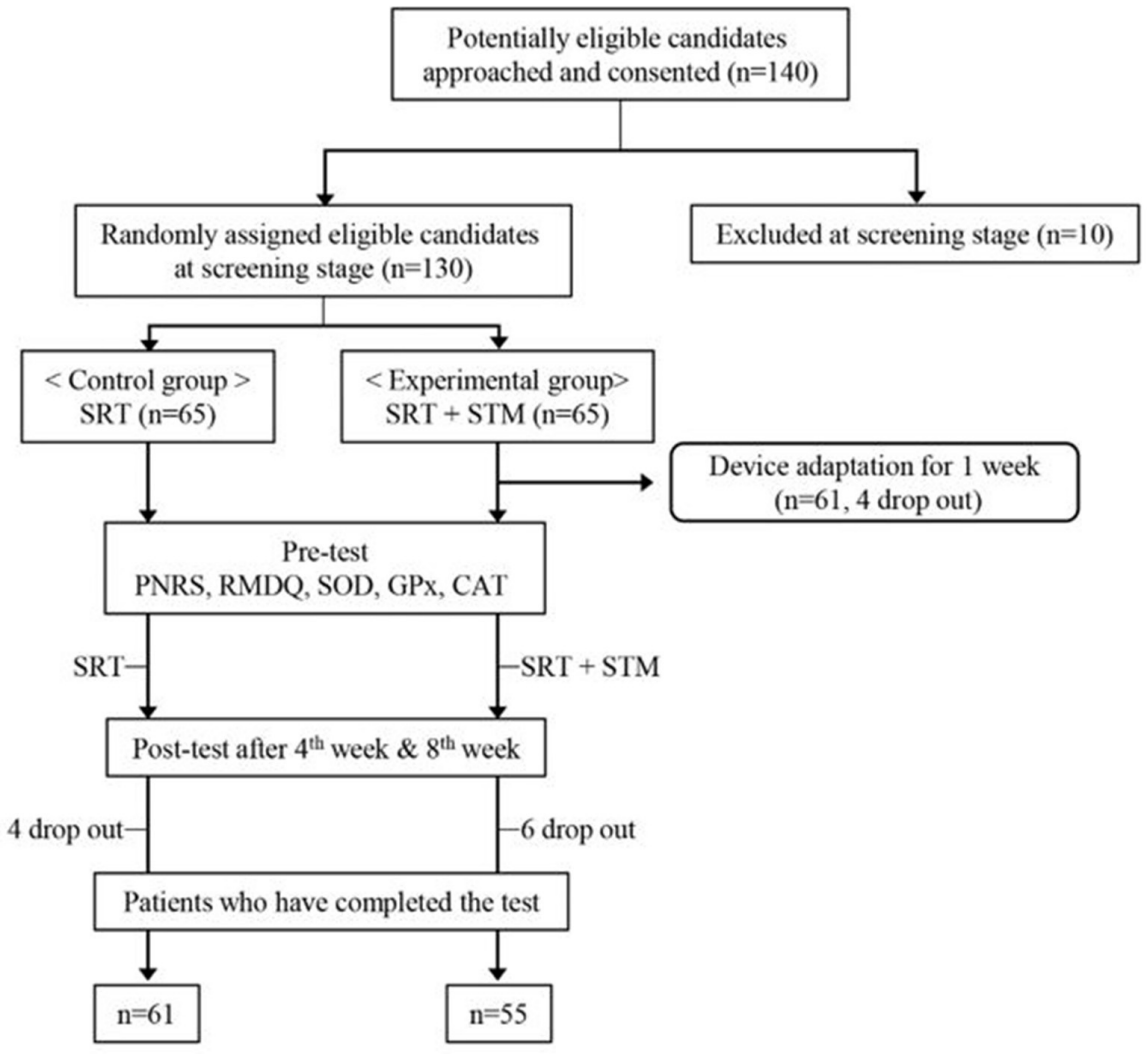
Study procedures.

Finally, a total of 130 participants were enrolled in the study. Sixty-five participants were assigned to the control group who received the standard rehabilitative treatment (SRT), whereas the remaining 65 patients were assigned to the experimental group who received SRT and a STM intervention. Participants were restricted from taking medications or nutraceutical foods that could affect the SOD, GPx, and CAT concentrations in the blood, which are used to measure the antioxidant enzyme activity. Given that some patients dropped out from the study, data of 116 participants (61 and 55 in the control and experimental groups, respectively) were ultimately used for the final analysis (Figure 1). There was no difference in the age distribution and pain duration between the two groups (Table 1). Although the sex distribution was different between the two groups (χ^2^ = 10.13, *p* < 0.05), a previous study showed that the effect of massage is not affected by sex difference (21).

**Table 1:** General characteristics of the participants.

**Variable**	**CON**	**EXP**	**Chi^**2**^ test**
**Sex**			
Male	23	37	*χ^2^* = 10.13
Female	38	18	*p* < 0.05
**Age (Years)**			
−69	27	32	*χ^2^* = 2.38
70–79	30	21	*p* > 0.05
80–	4	2	
**Pain Duration (Months)**			
1–3	7	9	
4–6	10	16	*χ^2^* = 4.24
7–12	16	11	*p* > 0.05
13–24	10	11	
25–	13	9	

### Massage Device

**Figure 2:**
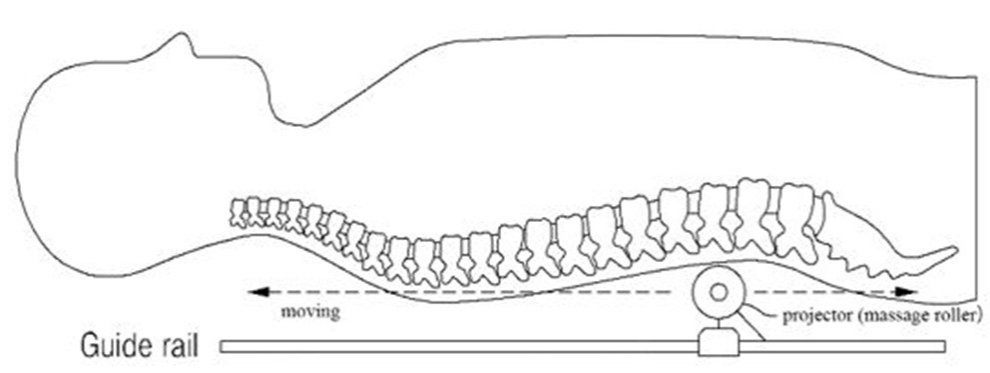
Schematic diagram of the spinal thermal massage device.

The present study used a thermal massage device that was approved as a personal medical device (Model: CGM MB-1401, Ceragem, South Korea). It consists of a bed covered with a tough synthetic fabric and a massage roller (projector) moving beneath the fabric. This device is operated with a remote control and allows the user to receive massage and heat treatment while lying down (Figure 2). The temperature of the projector is adjustable within the range of 30–60°C, and the strength of the massage ranged from level 1–6. The duration of the massage session set in this device is 36.5 min.

### Massage Intervention

Participants were instructed to lie on the device and operate it using the remote control. Before the actual massage, scanning is performed, wherein the projector moves from the cervical vertebrae to the sacrum, to measure the length and curvature of the spinal column. The participants were allowed to use the device 3–4 times for 1 week to become acclimatized. The STM intervention was applied five times per week for 8 weeks. Participants who experienced severe pain or discomfort during the massage session were excluded from the study.

### Measured Variables and Tools

The present study used self-report questionnaires to determine the level of disability due to the lower back pain and other pains. Blood samples were also collected from the participants to determine the antioxidant effects of the massage intervention. A questionnaire was administered and blood samples were collected before the intervention (PRE), immediately after 4 weeks of intervention (POST1), and immediately after 8 weeks of intervention (POST2).

#### Pain-Related Indices

The following two different clinical pain-related indices were measured: (1) the pain numeric rating scale (PNRS) and (2) the Roland and Morris disability questionnaire (RMDQ), which is used to determine the level of disability due to lower back pain. The PNRS is a segmented numeric version of the visual analog scale (VAS), in which a participant selects a whole number (0–10 integers) that best reflects the intensity of his/her pain (22). The common format is a horizontal bar or line. Similar to the VAS, the PNRS is anchored by terms describing pain severity extremes (23). The RMDQ is a 24-items self-report questionnaire about how low back pain affects functional activities. Each question is worth one point; thus, scores can range from 0 (no disability) to 24 (severe disability) (24). Higher scores for the PNRS and RMDQ indicate higher levels of pain and disability, respectively.

#### Measurement of Antioxidant Enzyme Activity

SOD, GPx, and CAT are representative antioxidants in the body that deal with free radicals that cause aging and disease. In the present study, blood samples were collected from the participants to determine the effects of the STM intervention on the antioxidant function based on the activities of SOD, GPx, and CAT.

#### Blood Sampling

Blood was collected before and immediately after 4 and 8 weeks of the massage intervention. The collected blood samples were immediately divided into serum collection tubes (BD Vacutainer® SST™ II Advance Plus Blood Collection Tube; Becton Dickinson UK Ltd., Oxford, UK) and heparinized tubes (BD Vacutainer® Sodium Heparin 75 USP Units Blood Collection Tube; Becton Dickinson UK Ltd.) for immediate transport to the laboratory.

#### Superoxide Dismutase

The SOD activity was measured using a SOD assay kit (Bioassay Systems, Hayward, CA, USA) according to the manufacturer's protocol. Briefly, the removal of superoxide by SOD was indirectly evaluated by measuring the reduction in cytochrome c levels. The protocol is as follows: First, the standards and samples were prepared to measure the SOD activity. Then, 160-μL assay buffer, 5-μL xanthine, and 5-μL WST-1 were mixed in each well. Subsequently, 160-μL working reagent was transferred to each well, the tap plate was mixed, XO enzyme was immediately added after reading OD440nm (ODo), and the plates were incubated for 60 min. Finally, we read the OD440nm (OD_60_) again to obtain the changed SOD value.

#### Glutathione Peroxidase

The GPx activity was measured using a GPx assay kit (Bioassay Systems) according to the manufacturer's protocol and was calculated based on the reduction of nicotinamide adenine dinucleotide phosphate. For GPx measurements, we prepared enough working reagents for all sample and control wells by mixing, for each well, 90-μL assay buffer, 5-μL glutathione, 3-μL 35-mM nicotinamide adenine dinucleotide phosphate, and 2-μL GR enzyme. We quickly added 90 μL of a working reagent to the sample/control wells. Then, this solution was diluted in dH2O with a 1:10 ratio to generate the substrate solution. The diluted solutions were used within 1 h. Using a multi-channel pipettor, we added a 100-μL substrate solution to the sample and control wells and mixed the contents well. We immediately read OD340nm (time 0, OD_0_) and read again in 4 min (OD_4_).

#### Catalase

CAT catalyzes the decomposition of hydrogen peroxide (produced by SOD) to oxygen and water. The CAT activity was measured using a CAT assay kit (Bioassay Systems) according to the manufacturer's instructions. The protocol is as follows. We prepared enough detection reagent by mixing, for each reaction well (sample, control, and standard wells), 102-μL assay buffer, 1-μL dye reagent, and 1-μL HRP enzyme. After completion of incubation for 30 min, a 100-μL detection reagent was added and mixed, followed by incubation for 10 min. Then, we read at 570 nm.

### Statistical Analysis

PNRS and RMDQ scores were analyzed by non-parametric methods, using the Mann–Whitney *U* and Freedman tests, because they are measured with ordinal scales. We used a mixed design with three-repeated measurements (PRE, POST1, and POST2). Accordingly, a two-way repeated measure analysis of variance was conducted for the statistical analysis of the data (SOD, GPx, and CAT). After omnibus testing, we analyzed the differences in the simple main effects of the interventions within the two groups and between the two groups at each time point. A *p* < 0.05 was considered statistically significant.

## Results

### Pain-Related Indices

The medians and interquartile ranges of PNRS and RMDQ scores are presented in Table 2. The Mann–Whitney *U*-test and Freedman test were applied to analyze the intergroup differences at each measurement time and intergroup differences in each group, respectively. The Freedman test showed that the pain levels decreased significantly in both the control (χ^2^ = 8.74, df = 2, *p* < 0.05) and experimental (χ^2^ = 68.88, df = 2, *p* < 0.01) groups. The Mann–Whitney *U*-test showed that the pain level was higher in the experimental group than in the control group before the intervention (*U* = 973.00, *p* < 0.01) and at POST1 (*U* = 1282.50, *p* < 0.05). Pain levels were reversed at POST2 with higher pain levels in the control group than in the experimental group (*U* = 1269.50, *p* < 0.05). These analyses indicate that pain reduction was greater in the experimental group than in the control group.

**Table 2:** Effect of spinal thermal massage on PNRS and RMDQ scores.

	**Group**	**PRE**	**POST1**	**POST2**
PNRS	CON (*n* = 61)	3.00 (1)	3.00 (2)	3.00 (2)
	EXP (*n* = 55)	4.00 (2)^##^	4.00 (2)^#^	2.00 (2)^#^
RMDQ	CON (*n* = 61)	6.00 (3)	6.00 (3)	6.00 (4)
	EXP (*n* = 55)	11.00 (6)^##^	9.00 (7)^##^	8.00 (9)^##^

*Data are expressed as median (interquartile range)*.

# (p < 0.05) and

## *(p < 0.01) indicate mean significant differences (Mann–Whitney U-test)*.

*PNRS, pain numeric rating scale; RMDQ, the Roland and Morris Disability Questionnaire; PRE, pre-intervention; POST1, 4 weeks post-thermal massage treatment; POST2, 8 weeks post-thermal massage treatment; CON, control; EXP, experimental*.

The Mann–Whitney *U*-test showed that the RMDQ scores were higher in the experimental group than in the control group before the intervention (*U* = 538.00, *p* < 0.01), at POST1 (*U* = 948.00, *p* < 0.01), and at POST2 (*U* = 1162.00, *p* < 0.01). The Freedman test showed that the RMDQ scores decreased in the experimental group as time progressed (χ^2^ = 43.19, df = 2, *p* < 0.01); however, the RMDQ scores in the control group did not. The results of these analyses indicate that the disability improved in both groups, but the degree of improvement was higher in the experimental group.

### Antioxidant Enzyme Activity

The SOD activity was significantly affected by the intervention [F_(1,114)_ = 3.97, *p* < 0.05] and measurement time [F_(2,228)_ = 8.56, *p* < 0.01]. There was a significant interaction effect on SOD activity [F_(2,228)_ = 10.04, *p* < 0.01]. When simple main effects were analyzed to examine these differences in more detail, there were no significant differences in the SOD activity between pre-intervention and at POST1 and POST2 in the control group. However, the SOD activity was significantly higher at POST1 [F_(1,54)_ = 13.76, *p* < 0.01] and POST2 [F_(1,54)_ = 32.53, *p* < 0.01) than before the treatment in the experimental group. There were no significant differences in the SOD activity between the two groups before the intervention, but the SOD activity was significantly higher in the experimental group at POST1 [F_(1,114)_ = 5.78, *p* < 0.05) and POST2 [F_(1,114)_ = 18.11, *p* < 0.01).

The intervention and measurement time had no significant effects on GPx activity, but there was a significant interaction effect observed for time and intervention on GPx activity [F_(2,228)_ = 9.58, *p* < 0.01]. The GPx activity was significantly lower at POST2 in the control group [F_(1,60)_ = 4.316, *p* < 0.05], whereas the GPx activity was significantly higher at POST2 in the experimental group [F_(1,54)_ = 13.68, *p* < 0.01]. The levels of GPx activity were not significantly different between the two groups before the intervention and at POST1, but the GPx activity was significantly higher in the experimental group at POST2 [F_(1,114)_ = 14.13, *p* < 0.01].

**Figure 3:**
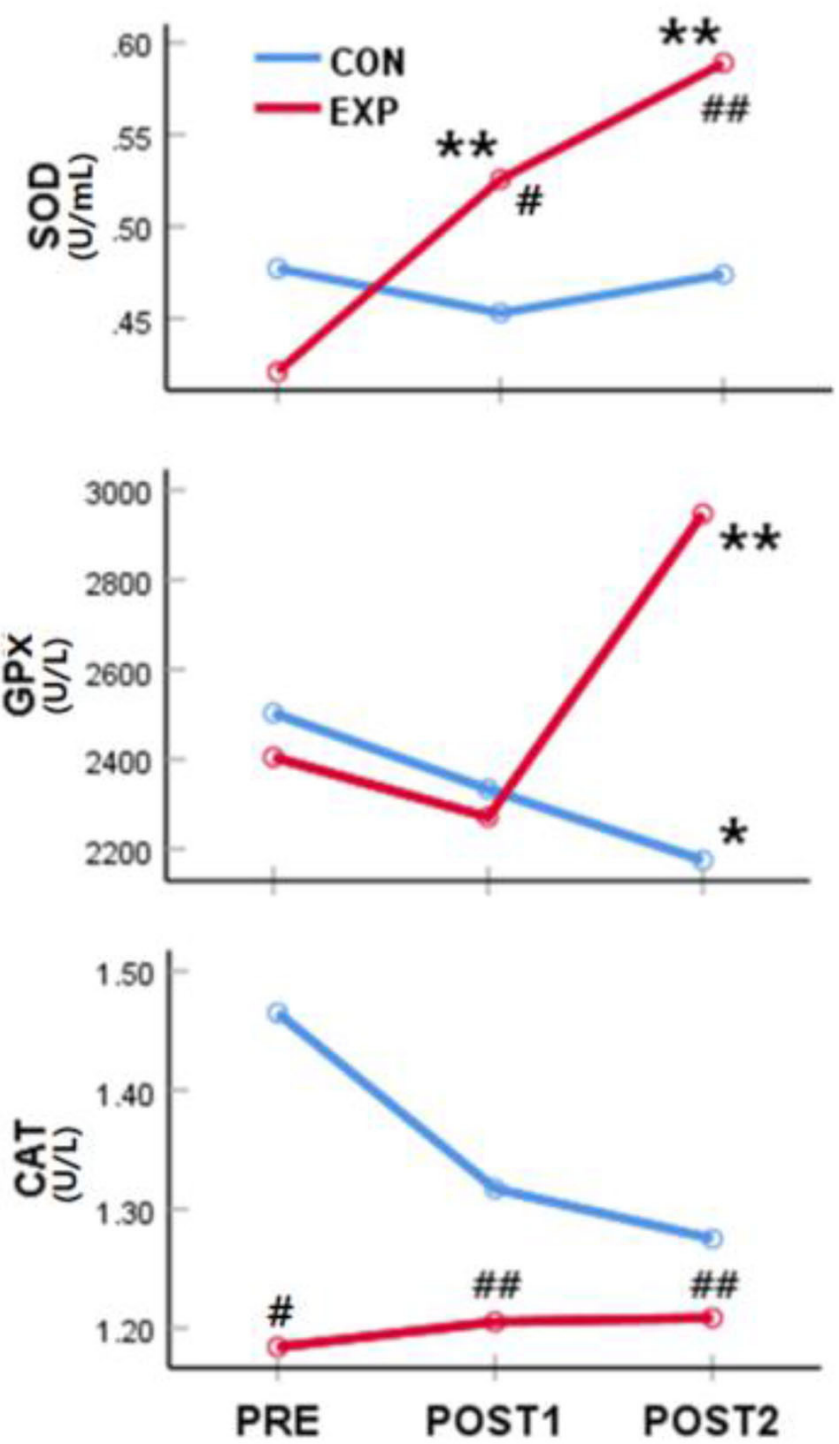
Effect of the spinal thermal massage on antioxidant function. *significant difference from “PRE” values (*p* < 0.05); **significant difference from “PRE” values (*p* < 0.01); ^#^significant difference from the control group (*p* < 0.05). ^##^significant difference from the control group (*p* < 0.01). PRE, pre-intervention; POST1, 4 weeks post-thermal massage treatment; POST2, 8 weeks post-thermal massage treatment; SOD, superoxide dismutase; GPx, glutathione-peroxidase; CAT, catalase; CON, control; EXP, experimental.

Overall, the CAT activity was higher in the experimental group than in the control group [F_(1,114)_ = 10.88, *p* < 0.01]; however, the interaction effect of time and effect was not significant. When the simple main effects were analyzed to examine these differences in more detail, there were no changes in the CAT activity before and after treatment in both groups. However, the CAT activity was significantly higher in the control group than in the experimental group before the intervention [F_(1,114)_ = 4.51, *p* < 0.05] and at POST1 [F_(1,114)_ = 41.04, *p* < 0.01] and POST2 [F_(1,114)_ = 9.09, *p* < 0.01] (Table 3, Figure 3). These results demonstrated that the CAT activity, which was higher in the control group than in the experimental group before the intervention, was maintained without any changes after 4 and 8 weeks of treatment.

**Table 3:** Effect of spinal thermal massage on antioxidant function.

	**Group**	**PRE**	**POST1**	**POST2**
SOD	CON (*n* = 61)	0.48 (0.02)	0.45 (0.02)	0.47 (0.02)
(U/mL)	EXP (*n* = 55)	0.42 (0.03)	0.53 (0.02)^**#^	0.59 (0.02)^**##^
GPx	CON (*n* = 61)	2502.87 (114.01)	2333.36 (117.87)	2174.84 (132.16)^*^
(U/L)	EXP (*n* = 55)	2404.44 (116.13)	2270.84 (111.53)	2946.71 (159.05)^**^
CAT	CON (*n* = 61)	1.46 (.012)	1.32 (0.01)	1.28 (0.01)
(U/L)	EXP (*n* = 55)	1.18 (0.02)^#^	1.21 (0.02)^##^	1.21 (0.02)^##^

*Values are expressed as the mean and standard error (SE)*.

* *Significant difference from “PRE” values (p < 0.05)*.

** *Significant difference from “PRE” values (p < 0.01)*.

# *Significant difference from the control group (p < 0.05)*.

## *Significant difference from the control group (p < 0.01)*.

*PRE, pre-intervention; POST1, 4 weeks post-thermal massage treatment; POST2, 8 weeks post-thermal massage treatment; SOD, superoxide dismutase; GPx, glutathione-peroxidase; CAT, catalase; CON, control; EXP, experimental*.

### Correlations Between Pain and Antioxidant Activity

To investigate the correlations between changes in antioxidant activity and pain-related measurements following the massage intervention, we performed a correlation analysis using the results from the antioxidant and pain analyses of the experimental group. The rate of change in values measured before and after the massage intervention was calculated and applied to the correlation analysis [rate of change (%) = 100 ^*^ (measured value after massage – measured value before massage) / measured value before massage]. With respect to the rate of change at POST1, the analysis revealed that the correlations between changes in pain and the values of the antioxidation indices were all significant (*p* < 0.05). Additionally, with respect to the rate of change at POST2, the correlations between changes in PNRS scores and the values of the antioxidation indices, except for CAT activity, were significant (*p* < 0.05) (Table 4).

**Table 4:** Correlations between the change rates in pain-related indices and antioxidant activity.

		**PNRS**	**RMDQ**
Change rate at POST1	SOD	0.870^*^	0.880^*^
	GPx	0.440^*^	0.322^*^
	CAT	0.229^*^	0.346^*^
Change rate at POST2	SOD	0.988^*^	0.987^*^
	GPx	0.517^*^	0.598^*^
	CAT	0.162	0.371^*^

* *p < 0.05*.

*PNRS, pain numeric rating scale; RMDQ, Roland and Morris disability questionnaire; SOD, superoxide dismutase; GPx, glutathione peroxidase; CAT, catalase*.

## Discussion

To date, only a few studies have attempted to monitor the changes in antioxidant enzyme levels and pain by simultaneously using massage and heat as sources of stimuli. This is the first study wherein two stimuli were used to confirm the increase in the antioxidant enzyme levels and the decrease in pain intensity in the elderly with reduced antioxidant enzyme levels. The subjective levels of pain (PNRS) and disability in performing activities of daily living due to lower back pain (RMDQ) were significantly lower (improved) in the experimental group than in the control group after SRT with thermal massage. Antioxidant function, as measured by the activities of SOD and GPx, was also improved following SRT with thermal massage compared to that observed after SRT only. This finding is consistent with the results of other studies that reported that the production of SOD and the level of glutathione (GSH), which is involved in GPx activity, were increased following massage (16), and that antioxidant enzyme levels were increased after thermal stimulation (25). We also observed a positive correlation between the extent of improvement in pain-related indices (PNRS and RMDQ) and antioxidant function, which suggests that the thermal massage intervention affected the physiological processes associated with pain and antioxidant enzyme activities.

Muscle pain is a complex progressive health problem that causes muscle weakness and fatigue (26). There are various causes of pain, including strenuous exercise or musculoskeletal injury, inflammatory response to oxidative stress, including ROS, and fatigue due to lactic acid build-up (27). Watkins et al. suggested that inflammatory cytokines, including IL-1ß, which are released from the microglial cells in the peripheral and central nervous system (CNS), are associated with pain, and that ROS can cause chronic pain by activating the glial cells in the CNS (28). Perez et al. reported that the symptoms of complex regional pain syndrome type I (CRPS-I) were reduced after the administration of ROS scavengers (29). This finding also serves as evidence that antioxidant activity is associated with pain. Based on the results of these studies and the correlation analysis in the present study, we believe that antioxidant function is improved by thermal massage. Consequently, pain alleviation is also achieved with improved antioxidant function, which protects against ROS, and this antioxidant function appears to decrease with aging. The concentrations of hepatic GSH and glutathione reductase, and the activity of SOD have been found to be lower in older animals than in younger animals (30). Even in humans, compared to the levels of antioxidant enzymes in 25-years-old individuals, these levels decrease by 30, 40, and 60% in people aged 40–49, 60–69, and 70–79 years, respectively (31). The SOD activity has been found to decrease with age, with the metabolism of antioxidants consumed through food also decreasing (32, 33). The level of antioxidant enzyme, SOD, which has the ability to remove ROS, increases when ROS and lipid peroxide are generated in the body. This ability rapidly decreases after the age of 40 years, which can lead to various geriatric diseases (34, 35). Diabetes and lung cancer related to smoking rarely develop in people with high SOD activity. However, if the SOD activity decreases, the probability of developing brain and cardiovascular disorders, such as cancer, stroke, and myocardial infarction, increases (34).

GPx catalyzes the decomposition of hydrogen peroxide formed by SOD into oxygen and water, and promotes the activity of GSH, which catalyzes the decomposition and detoxification of lipid peroxides (36). GPx plays a secondary role in removing the remaining hydrogen peroxide after the action of CAT on hydrogen peroxide (37). Low GPx activity can lead to exposure to several diseases, including multiple sclerosis and diabetic kidney disease. Various reports have indicated that the ability to synthesize antioxidant enzymes, efficiency of antioxidant enzymes, and relevant physiological metabolic actions all decrease with age, clearly suggesting that antioxidant function should be augmented in elderly populations. The mean age of the participants in the present study was 65 years, and improvement in their antioxidant function was achieved through an intervention involving a thermal massage device.

We recognize some limitations associated with the study presented here. First, although we identified that the use of thermal massage improved the antioxidant function, we did not evaluate the specific mechanisms involved in this improvement. Second, as the role of antioxidant function in aging and various diseases has already been established, additional studies should investigate the effects of thermal massage on improving other symptoms or other diseases associated with antioxidant function. Finally, future studies should investigate whether a thermal massage program affects the improvement in antioxidant function for various causes of back pain, such as stress, exercise, injury, or inflammatory responses.

## Conclusion

While additional systematic studies are required, the findings of this study suggest that thermal massage may be an effective strategy for reducing pain and preventing a decrease in antioxidant enzyme activity in elderly individuals. Thermal massage may, therefore, be useful in the prevention and treatment of diseases associated with oxidation. No side effects due to the intensity of massage and heat stimulation were reported during this trial.

## Data Availability Statement

All data generated for this study are included in the article.

## Ethics Statement

The studies involving human participants were reviewed and approved by Institutional Review Board at the Presbyterian (Jesus) Medical Center (IRBN. 2017-06-022). The patients/participants provided their written informed consent to participate in this study.

## Author Contributions

K-EK: conception, design, material preparation, data collection, analysis, writing—original draft, review, and editing. I-YC: conception, design, material preparation, data collection, analysis, review writing, editing, and project administration. Y-SY and S-YN: conception, design, material preparation, data collection, and analysis. S-KP: conception, design, material preparation, data collection, analysis, review writing, and editing. J-SP: conception, design, material preparation, data collection, analysis, review writing, editing, and supervision. All authors contributed to the article and approved the submitted version.

## Conflict of Interest

The authors declare that the research was conducted in the absence of any commercial or financial relationships that could be construed as a potential conflict of interest.
